# Supplementary material for: Splice-Junction-Based Mapping of Alternative Isoforms in the Human Proteome
Source: Cell Rep. Author manuscript; Available in PMC 2020 Jan 15. (PMC6961840; doi:10.1016/j.celrep.2019.11.026)
Supplement: 2 [file NIHMS1546469-supplement-2.zip › DF1/PXD010154/Ovary/Ovary_16_RFC1_TTTASLVCQMK.pdf]

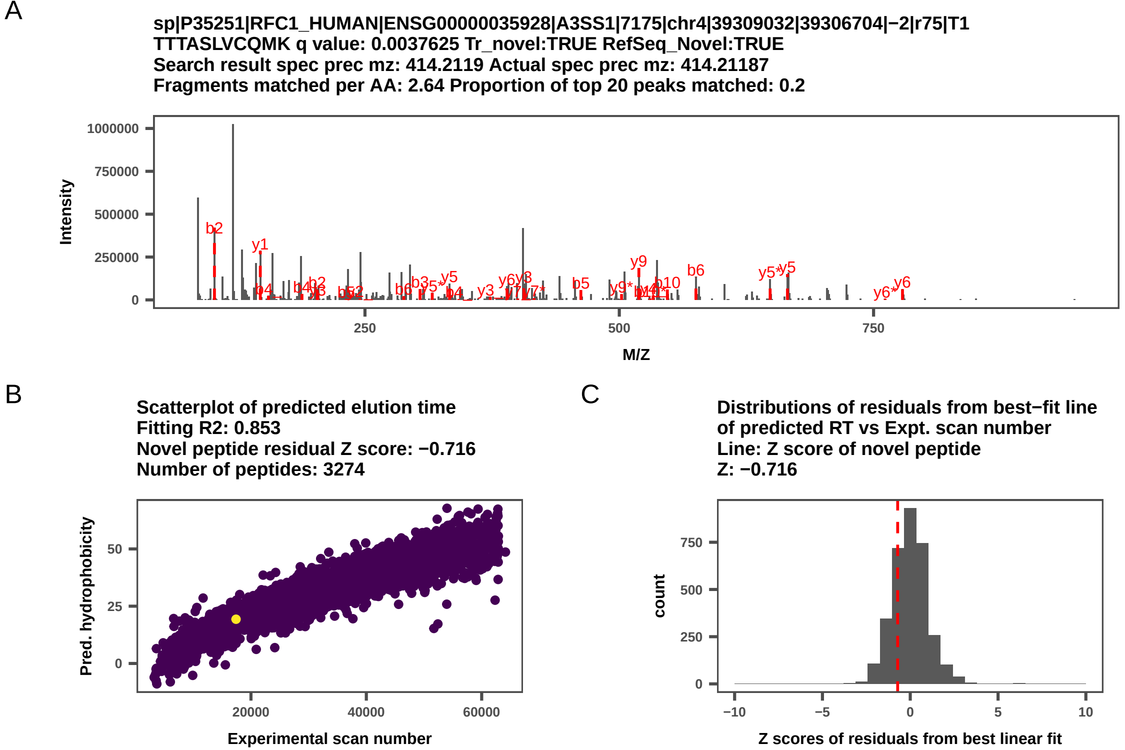
A
sp|P35251|RFC1_HUMAN|ENSG00000035928|A3SS1|7175|chr4|39309032|39306704|−2|r75|T1
TTTASLVCQMK q value: 0.0037625 Tr_novel:TRUE RefSeq_Novel:TRUE
Search result spec prec mz: 414.2119 Actual spec prec mz: 414.21187
Fragments matched per AA: 2.64 Proportion of top 20 peaks matched: 0.2
Intensity
1000000
750000
500000
250000
0
b2
y1
y9
b6
y5*
y5
b10
y6
y6*
b5
y9*
y5
y3
b4
M/Z
250
500
750
B
Scatterplot of predicted elution time
Fitting R2: 0.853
Novel peptide residual Z score: −0.716
Number of peptides: 3274
Pred. hydrophobicity
50
25
0
20000
40000
60000
Experimental scan number
C
Distributions of residuals from best−fit line
of predicted RT vs Expt. scan number
Line: Z score of novel peptide
Z: −0.716
count
750
500
250
0
−10
−5
0
5
10
Z scores of residuals from best linear fit
